# Investigation of the correlation between serum levels of interleukin-6 and clinical and paraclinical findings in COVID-19 patients

**DOI:** 10.1016/j.curtheres.2026.100847

**Published:** 2026-09-26

**Authors:** Hossein Dalilgo, Zahra Soleimani, Kamal Esalatmanesh, Hamidreza Gilasi, Javad Amini Mahabadi, Atena Abed

**Affiliations:** a Infectious Diseases Research Center, Kashan University of Medical Sciences, Kashan, Iran; b Internal Medicine Department, Kashan University of Medical Sciences, Kashan, Iran; c Department of epidemiology and Biostatistics, Heath faculty, Kashan University of Medical Science, Kashan, Iran; d Anatomical Sciences Research Center, Institute for Basic Sciences, Kashan University of Medical Sciences, Kashan, Iran; e Department of Medical Biotechnology, School of Advanced Technologies, Shahrekord University of Medical Sciences, Shahrekord, Iran

**Keywords:** Clinical study, COVID-19 testing, Demographic factors, Interleukin 6, Observational studies

## Abstract

**Background:**

COVID-19 frequently presents with pneumonia. Impaired immune function, particularly in older adults and immunocompromised individuals, increases susceptibility to infection and contributes to higher morbidity and mortality. Among inflammatory cytokines, Interleukin-6 (IL-6) plays a pivotal role in immune dysregulation and cytokine storm. Therefore, this study aimed to investigate the association between serum IL-6 levels and clinical, laboratory, radiological, and outcome-related findings among hospitalized patients with COVID-19.

**Methods:**

This descriptive-analytical cross-sectional study included 210 hospitalized COVID-19 patients at Shahid Beheshti Hospital, Kashan. Serum IL-6 was measured as part of their diagnostic and therapeutic workup. Demographic characteristics, clinical manifestations, laboratory markers, imaging findings, and outcomes were recorded. Patients with incomplete data were excluded. Data were analyzed using SPSS software version 26.

**Results:**

The mean age of patients was 50.37 ± 17.48 years, with a slight male predominance (51%). Hypertension was the most common comorbidity (31.9%). Abnormal laboratory markers included elevated LDH (64.3%), ESR (61.0%), lymphopenia (59.5%), and CRP (58.6%). Pulmonary involvement on CT was noted in 91.5% of patients, with moderate involvement most frequent (41.4%). ICU admission occurred in 17.1% and mechanical ventilation was required in 10.5%. The mean CALL score was 8.63 ± 2.33, and average hospital stay was 6.70 ± 5.27 days. Serum IL-6 levels were significantly associated (*P* < 0.05) with age, oxygen saturation, lymphopenia, LDH, CRP, CT involvement, disease severity, ICU admission, mortality, and length of hospitalization.

**Conclusion:**

Elevated serum IL-6 strongly reflects disease severity and adverse outcomes in COVID-19, highlighting its potential as a prognostic biomarker to guide clinical assessment and management.

## Graphical abstract

Correlation between serum interleukin-6 (IL-6) levels and clinical, laboratory radiological, and outcome parameters in hospitalized COVID-19 patients (This figure was designed by FigureLab).

**Figure:**
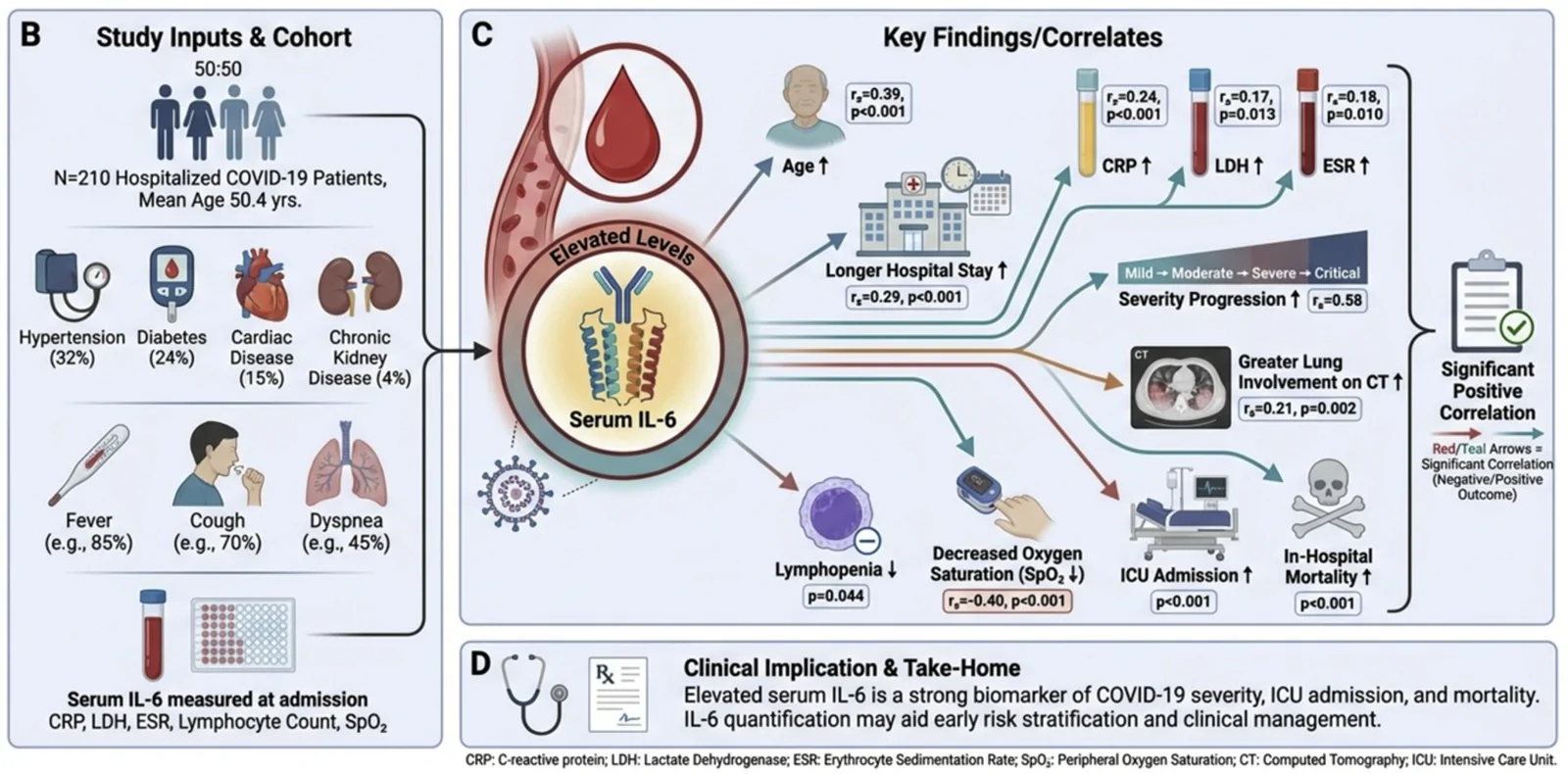

**Abbreviations**
COVID-19: Coronavirus Disease
SARS-CoV-2: Severe Acute Respiratory Syndrome Coronavirus 2
Ct Scans: Computed Tomography Scan
LDH: levels Lactate Dehydrogenase
ESR: Erythrocyte Sedimentation Rate
CRP: C-reactive protein
CALL: score Comorbidity‐Age‐Lymphocyte count‐Lactate dehydrogenase
WHO: World Health Organization
TMPRSSs: Transmembrane Serine Proteases
STAT3: Signal Transducer and Activator of Transcription 3
ACE2: Angiotensin-Converting Enzyme 2
RT-PCR: Reverse Transcription Polymerase Chain Reaction
IL-6: Interleukin-6
IL-6R: Interleukin-6 Receptor
SAA: Serum Amyloid A

## Introduction

On 31st December 2019, the authorities in China informed about the rising incidence of severe pneumonia in the city of Wuhan, Hubei province in China. They confirmed identifying for the first time a new coronavirus, which is a family of microRNA respiratory viruses. The new virus was temporarily referred to as the virus "2019-nCoV".^1^ In March 2020, the World Health Organization (WHO) classified it as a pandemic, which has caused world-wide wide-spread morbidity and mortality since then. To date (December 2022), the disease has caused an estimated 6.6 million deaths and infection in over 651 million people.^2^ 2019-nCoV belongs to the subfamily Coronavirinae in the family of Coronaviridae of the order Nidovirales, and this subfamily includes four genera: Alphacoronavirus, Betacoronavirus, Gammacoronavirus, and Deltacoronavirus. The genome of 2019-nCoV is a single‐stranded positive‐sense RNA.^3^

Hospitalized patients have common symptoms such as dry cough, fever, shortness of breath nausea, fatigue, diarrhea, and myalgia vomiting. Gastrointestinal signs are also atypical symptoms in patients.^4^, ^5^, ^6^ On the host cell there is a receptor named angiotensin-converting enzyme 2 (ACE2) that coronavirus binds to it by its spike (S) protein. Transmembrane serine proteases (TMPRSSs) on the host cell cleavages spike (S) protein and lets the virus releasing fusion peptides to attach the membrane.^7^ Development and utilization of diagnostic methods in laboratory for detection of viral antigens or antibodies (Ig-G and Ig-M) have been increased.^8^ Effective and new diagnostic strategies such as RT-PCR, have been used to monitor this disease.^9^ Nevertheless, The diagnostic methods do not have 100% sensitivity.^10^ Detection of viral antigen in nasal or throat samples is an important laboratory criterion, as other tests cooperate to diagnose or predicting severity of the disease.^11^ The most common findings in the laboratory between patients hospitalized with COVID-19 are lymphopenia, increased levels of lactate dehydrogenase, aminotransferase, inflammatory biomarkers (e.g., C-reactive protein, erythrocyte sedimentation rate and ferritin) and irregularities in blood clotting assessments.^12^ It has been displayed that cytokine storm plays a critical role in the progression to severe COVID-19. Relying on exist search, cytokines have central function in the pathological basis of COVID-19; even though several of them are advantageous, others emerge detrimental like interleukin-6 mainly during the cytokine storm.^13^

Indeed, multiple diseases that are infectious or noninfectious can be lead to cytokine storms and damage to some organ.^14^ When IL-6 are overproduced and signaling pathways of IL-6 deregulates, I can lead to inflammation and impact in the network of human cytokine.^15^ Interleukin-6 (IL-6) has pro-inflammatory features and functions as a bioactive protein.^16^ Several immune and a few non-immune cells like fibroblasts produce IL-6.^17^ Transmembrane receptor (IL-6R), recognizes IL-6 with formation a complex with glycoprotein 130 (gp130). This receptor with tyrosine kinase activation starts phosphorylation to initiate signal transducer and activator of transcription 3 (STAT3).^18^ ADAM-17 protease cleaves the extracellular part of IL-6R from the membrane receptor. The soluble part of IL-6R that does not have activation of tyrosine kinase connects with gp130. Outside the cell, forms a complex with IL-6 and IL-6R for binding to the cell membrane. Cell type and interaction with receptor type impact on IL-6-IL-6R axis's functional.^19^ IL-6 positively has effective to maturate of B lymphocytes and cytotoxicity of T lymphocytes.^16^ In opposition, lack of IL-6 in investigational models care for autoimmune encephalomyelitis stimulation.^20^ Severe COVID-19 patients with showed advanced IL-6/IFN ratios rather patients who had mild disease, possibly because of a cytokine storm that is stronger leads to lung damage.^21^ This issue increases a question that if inhibition of IL-6 is advantageous entirely for persons with advanced serum IL-6 expression.^22^ Excluding for some exceptions, various studies displayed a correlation among elevated levels of CRP and severe COVID-19.^23^ Future study interested in biomarkers for risk arrangement and observing therapeutic results will be vital.^24^ Related clinical trials found no maintenance for the theory that utilizing tocilizumab (monoclonal antibody) that is anti-receptor for IL-6, could progress COVID-19 signs, for example hypoxia, respiratory failure, or decrease mortality. Nevertheless, tocilizumab might still be successful in severe patients, requiring further research.^25^ It have been shown that levels of Inflammatory cytokine to be significantly upper in cases with severe COVID-19 than in mild ones, and IL-6 is fundamental to the progression of severe inflammatory states. Therefore, this study aimed to investigate the association between serum IL-6 levels and demographic, clinical, laboratory, radiological, and clinical outcome-related variables, including disease severity and in-hospital outcomes, among hospitalized patients with COVID-19.

## Materials and methods

### Study design and population

This cross-sectional, descriptive-analytical study was conducted on patients with confirmed COVID-19 admitted to Shahid Beheshti Hospital in Kashan, Iran, between March 5, 2020, and March 20, 2023. A consecutive sampling method was utilized to include all eligible hospitalized patients during the study period. Inclusion criteria comprised: (1) definitive diagnosis of COVID-19 confirmed by reverse transcription-polymerase chain reaction (RT-PCR) from nasopharyngeal/oropharyngeal swabs according to World Health Organization (WHO) interim guidance; (2) age ≥ 14 years; and (3) available baseline serum Interleukin-6 (IL-6) measurements obtained at the time of admission. Patients younger than 14 years, pregnant individuals, and those with incomplete clinical, laboratory, or outcome records were excluded. Ultimately, a total of 210 validated patient records were included in the final analysis.

### Clinical definitions and disease severity stratification

Patients were stratified into four distinct clinical severity categories upon admission according to the WHO clinical management guidelines and national COVID-19 protocols:

Mild: Symptomatic patients meeting the case definition for COVID-19 without evidence of viral pneumonia or hypoxia.

Moderate: Patients presenting with clinical signs of pneumonia (fever, cough, dyspnea, fast breathing) but without signs of severe pneumonia, maintaining an oxygen saturation (SpO_2_) ≥ 90% on room air.

Severe: Patients showing clinical signs of pneumonia plus at least one of the following: respiratory rate >30 breaths/min, severe respiratory distress, or SpO_2_ <90% on room air.

Critical: Patients exhibiting acute respiratory distress syndrome (ARDS), sepsis, septic shock, or other conditions requiring mechanical ventilation or intensive care unit (ICU) admission.

### Demographic and baseline characteristics

Age, gender, smoking status, and underlying comorbidities (e.g., hypertension, diabetes mellitus, cardiovascular disease, chronic kidney disease, and liver disease).

features and outcomes Clinical symptoms upon admission, requirement and modalities of oxygen therapy, ICU admission, total length of hospital stay (LOS), and disease severity stratification.
data Chest computed tomography (CT) scan findings evaluated by expert radiologists and categorized according to the extent of pulmonary involvement.
parameters Baseline laboratory tests performed at admission, including complete blood count components (WBC, neutrophil status, presence of lymphopenia, anemia, and platelet count), biochemical and inflammatory markers (erythrocyte sedimentation rate [ESR], C-reactive protein [CRP], lactate dehydrogenase [LDH], creatine phosphokinase [CPK], liver transaminases [AST, ALT], and cardiac troponin).

Venous blood samples were collected upon hospital admission as part of the routine baseline clinical workup, prior to the initiation of in-hospital therapeutic protocols. Baseline serum IL-6 concentrations were measured by the central hospital laboratory using standard quantitative enzyme-linked immunosorbent assay (ELISA) kits according to institutional protocols.

### Ethical considerations

The study protocol was reviewed and approved by the Institutional Ethics Committee of Kashan University of Medical Sciences (Approval Code: IR.KAUMS.MEDNT.REC.1401.029). The study complied fully with the ethical principles outlined in the Declaration of Helsinki. Written informed consent was obtained from all conscious patients or their legally authorized representatives. All patient data were anonymized and de-identified prior to statistical analysis to maintain confidentiality.

### Sample size determination

The minimum required sample size was calculated a priori using G*Power software (version 3.1.9.7) based on bivariate correlation analysis between serum IL-6 levels and clinical/laboratory parameters. Assuming a two-tailed significance level (α) of 0.05, a statistical power (1 − β) of 80%, and an anticipated moderate effect size (*r* = 0.20) derived from previous COVID-19 biomarker studies, the minimum required sample size was determined to be 194 patients. To account for potential missing data or exclusions, a total of 210 eligible patients were ultimately enrolled, providing adequate statistical power (>85%) to detect the observed correlations and subgroup differences*.*

### Statistical analyses

Statistical analyses were conducted using IBM SPSS Statistics for Windows, Version 26.0. Categorical variables are presented as frequencies and percentages (*n*, %). The normality of continuous variables was evaluated using the Kolmogorov–Smirnov and Shapiro–Wilk tests, along with visual inspection of histograms and Q-Q plots. Variables following a normal distribution are expressed as mean ± standard deviation (SD), whereas non-normally distributed continuous variables, including serum IL-6 levels, are summarized as median and interquartile range (IQR; 25th–75th percentiles). For categorical comparisons, the Pearson Chi-square test or Fisher’s exact test was applied as appropriate. For continuous and ordinal variables with non-normal distributions, the Mann–Whitney U test was used for two-group comparisons, and the Kruskal–Wallis test was employed for comparisons across multiple groups. Correlations between serum IL-6 levels and clinical, laboratory, and radiological parameters were assessed using Spearman’s rank correlation coefficient (*r_s_*). All tests were two-tailed, and a *P*-value of <0.05 was considered statistically significant.

## Results

In this study, 210 patients with confirmed COVID-19 who met the inclusion criteria were evaluated. Gender was evenly distributed across the cohort, with 50.0% male and 50.0% female patients. The most prevalent blood group was blood group O, accounting for 35.7% of the patients. Overall, 11.0% of patients had been vaccinated, and 9.5% had a history of previous COVID-19 infection. A history of smoking was documented in 17.1% of cases. The most frequent underlying comorbidities were hypertension (31.9%), diabetes mellitus (23.8%), cardiovascular disease (14.8%), and chronic kidney disease (4.3%). The most common presenting clinical symptoms were fever (68.6%), cough (61.4%), and dyspnea (53.8%) (Table 1). Continuous demographic and clinical parameters were summarized as mean ± standard deviation or median with interquartile range, as appropriate, whereas categorical variables were reported as frequencies and percentages. The baseline laboratory findings are presented in Table 2. The most prevalent paraclinical abnormalities included elevated lactate dehydrogenase (LDH) levels (64.3%), elevated erythrocyte sedimentation rate (ESR) (61.0%), lymphopenia (59.5%), and elevated C-reactive protein (CRP) levels (58.6%). Regarding radiological findings, 72.4% of patients had an abnormal chest X-ray, and 91.5% exhibited pulmonary involvement on chest computed tomography (CT) scans. Moderate pulmonary involvement was the most frequent CT pattern, occurring in 41.4% of patients (Table 1).

**Table 1:** Baseline characteristics of patients.

Variables	Frequency (%)	Mean ± Standard Deviation	
Age	-	50.37 ± 17.48	
Gender	Male	107 (51.0)	-
	Female	103 (49.0)	-
Blood Group	A+	49 (23.3)	-
	A-	11 (5.2)	-
	B+	43(20.5)	-
	B-	8 (3.8)	-
	AB+	16 (7.6)	-
	AB-	8 (3.8)	-
	O+	62 (29.5)	-
	O-	13 (6.2)	-
Vaccination History	23 (11.0)	-	
History of Infection with COVID-19	20 (9.5)	-	
Smoking	36 (17.1)	-	
Obesity	28 (13.3)	-	
Underlying Disease	High blood pressure	67 (31.9)	-
	Diabetes	50 (23.8)	-
	Lung disease	12 (5.7)	-
	Hypothyroidism	19 (9.0)	-
	Chronic Kidney Disease	3 (1.4)	-
	Heart disease	37 (17.6)	-
	Liver disease	8 (3.8)	-
	Rheumatologist disease	10 (4.8)	-
	Hyperlipidemia	26 (12.4)	-
Clinical Symptoms	Fever	144 (68.6)	-
	Myalgia	84 (40.0)	-
	Fatigue	86 (41.0)	-
	Cough	129 (61.4)	-
	Shortness of breath	113 (53.8)	-
	Sore throat	13 (6.2)	-
	Runny nose	18 (8.6)	-
	Diarrhea	27 (12.9)	-
	Loss of smell	7 (3.3)	-
	Loss of taste	7 (3.3)	-
	Headache	64 (30.5)	-
	Nausea/Vomiting	53 (25.2)	-
	Chills	85 (40.5)	-
	Excessive sweating	12 (5.7)	-
	Abdominal pain	12 (5.7)	-
	Loss of appetite	48 (22.9)	-
	Dizziness	11 (5.2)	-
	Hemoptysis (coughing up blood)	3 (1.4)	-
	Chest pain	35 (16.7)	-
White Blood Cell	Leukopenia (less than 4000)	-	19.5
	Normal	-	67.1
	Leukocytosis (greater than 11,000)	-	13.3
Absolute Neutrophil	Less than 2000	10 (4.8)	-
	Normal	167 (79.5)	-
	More than 8000	33 (15.7)	-
Lymphocytopenia (less than 1200)	125 (59.5)	-	
Anemia (Hemoglobin less than 12.5)	49 (23.3)	-	
Platelet	Less than 150 Thousand	69 (32.9)	-
	Normal	126 (60.0)	-
	More than 450 Thousand	15 (7.1)	-
CRP	Normal	87 (41.4)	-
	High	123 (58.6)	-
ESR	Normal	82 (39.0)	-
	More than 20	128 (61.0)	-
CPK	Normal	154 (73.3)	-
	High	56 (26.7)	-
LDH	Normal	75 (35.7)	-
	High (More than 550)	135 (64.3)	-
AST	Normal	138 (65.7)	-
	High (More than 40)	72 (34.3)	-
ALP	Normal	206 (98.1)	-
	High (More than 306)	4 (1.9)	-
ALT	Normal	171 (81.5)	-
	High (More than 40)	39 (18.6)	-
D Dimer	Normal	156 (74.3)	-
	High	54 (25.7)	-
TROP	Negative	205 (97.6)	-
	Positive	5 (2.4)	-
Level of Interleukin-6	-	1.82 ± 2.77	

CRP: C-reactive protein; ESR: Erythrocyte Sedimentation Rate; CPK: Creatine Phosphokinase; LDH: Lactate Dehydrogenase; AST: Aspartate Aminotransferase; ALP: Alkaline Phosphatase; ALT: Alanine Transaminase: TROP: Troponin.

**Table 2:** Frequency distribution of patient tests.

Imaging Findings		Frequency	Mean ± Standard Deviation
Chest X-Ray	Normal	58 (27.6)	-
	Abnormal	152(72.4)	-
Chest CT Scan	Normal	20 (9.5)	-
	Mild Lung Involvement	55 (26.2)	-
	Moderate Lung Involvement	87 (41.4)	-
	Severe Lung Involvement	48 (22.9)	

CT scan: Computed Tomography Scan.

The distribution of disease severity categories, clinical characteristics, and patient outcomes is summarized in Tables 3 and 5. The cohort comprised 73 patients (34.8%) with mild disease, 81 patients (38.6%) with moderate disease, 20 patients (9.5%) with severe disease, and 36 patients (17.1%) with critical disease (Table 3). Overall, 36 patients (17.1%) required admission to the intensive care unit (ICU), 22 patients (10.5%) received invasive mechanical ventilation, and 14 patients (6.7%) died during hospitalization. The mean CALL score was 8.63 ± 2.33, and the mean length of hospital stay was 6.70 ± 5.27 days (Table 5). Length of hospitalization, age, and oxygen saturation percentage were statistically significant (Table 4).

**Table 3:** Distribution of disease severity and serum IL-6 levels among hospitalized COVID-19 patients (*N* = 210).

Disease Severity Category	Frequency, *n*(%)	Serum IL-6 (pg/mL), Median (IQR)	IL-6 Range (Min–Max)	Statistical Test	*P*-value
Mild	73 (34.8%)	0.090 (0.160)	0.00 – 4.70	Kruskal–Wallis	<0.001
Moderate	81 (38.6%)	0.140 (0.290)	0.01 – 5.12	H = 83.291	
Severe	20 (9.5%)	4.490 (3.630)	0.10 – 8.94	(*df* = 3)	
Critical	36 (17.1%)	5.905 (3.568)	0.85 – 10.23		
Total	210 (100.0%)	0.170 (3.513)	0.00 – 10.23		

Note: Values are reported as number (percentage) for categorical variables and median (interquartile range [IQR: 25th–75th percentiles]) for serum IL-6 concentrations due to skewed distribution.

*H*: Kruskal–Wallis test statistic; *df*: degrees of freedom; *P* < 0.05 indicates statistical significance.

**Table 4:** Examination of the relationship between the studied quantitative variables and the level of interleukin-6 in patients.

Quantitative Variables	Pearson Correlation Coefficient	*P*-Value
Length of Hospitalization	0.289	0.001
Age	0.326	0.001
Oxygen Saturation Percentage	−0.485	0.001

**Table 5:** Examining the relationship between the qualitative variables studied and the interleukin-6 levels in patients.

Qualitative Variable		*P*-Value
Gender	Female	0.272
	Male	
Lymphocytopenia	Yes	0.006
	No	
LDH	Normal	0.007
	High	
CRP	Normal	0.001
	High	
Intensity of the Disease	Mild	0.001
	Moderate	
	Severe	
	Critical	
Chest CT Scan Findings	Mild Lung Involvement	0.001
	Moderate Lung Involvement	
	Severe Lung Involvement	
Admission to the ICU	Yes	0.001
	No	
Mortality	Yes	0.001
	No	

LDH: Lactate Dehydrogenase; CRP: C-Reactive Protein; CT: Tomography Scan; ICU: Intensive Care Unit.

### Correlation and Association of Serum IL-6 with Clinical, Paraclinical, and Outcome Parameters

Non-parametric correlation and comparative analyses demonstrated statistically significant associations between baseline serum interleukin-6 (IL-6) concentrations and several demographic, clinical, laboratory, radiological, and prognostic parameters (Table 6).

**Table 6:** Non-parametric correlation and comparative analysis of serum interleukin-6 (IL-6) levels with demographic, clinical, Laboratory findings, and patient outcomes (*N* = 210).

Variable Category	Variable	Statistical Test	Correlation Coefficient / Test Statistic	95% Confidence Interval (CI)	*P*-value
Demographics & Comorbidities	Age (years)	Spearman Rank	rs = 0.390	[0.269, 0.499]	<0.001
Demographics & Comorbidities	Gender (Male vs. Female)	Mann–Whitney U	U = 5214.5	—	0.457
Demographics & Comorbidities	Smoking History	Mann–Whitney U	U = 1381.0	—	0.380
Demographics & Comorbidities	Hypertension	Mann–Whitney U	U = 3719.5	—	<0.001
Demographics & Comorbidities	Diabetes Mellitus	Mann–Whitney U	U = 2873.5	—	<0.001
Demographics & Comorbidities	Cardiovascular Disease	Mann–Whitney U	U = 2278.0	—	<0.001
Demographics & Comorbidities	Chronic Kidney Disease	Mann–Whitney U	U = 811.5	—	0.007
Demographics & Comorbidities	Chronic Liver Disease	Mann–Whitney U	U = 582.0	—	0.655
Clinical Presentation	Baseline SpO2 (%)	Spearman Rank	rs = −0.398	[−0.506, −0.278]	<0.001
Clinical Presentation	Fever	Mann–Whitney U	U = 4991.0	—	0.812
Clinical Presentation	Cough	Mann–Whitney U	U = 5022.5	—	0.449
Clinical Presentation	Dyspnea	Mann–Whitney U	U = 4699.5	—	0.287
Inflammatory & Lab Markers	C-Reactive Protein (CRP)	Spearman Rank	rs = 0.238	[0.105, 0.362]	<0.001
Inflammatory & Lab Markers	Troponin	Spearman Rank	rs = 0.201	[0.067, 0.328]	0.003
Inflammatory & Lab Markers	ESR	Spearman Rank	rs = 0.177	[0.043, 0.306]	0.010
Inflammatory & Lab Markers	Lactate Dehydrogenase (LDH)	Spearman Rank	rs = 0.171	[0.036, 0.299]	0.013
Inflammatory & Lab Markers	Lymphopenia (Present vs. Absent)	Mann–Whitney U	U = 4310.5	—	0.044
Inflammatory & Lab Markers	Anemia (Present vs. Absent)	Mann–Whitney U	U = 4426.0	—	0.052
Inflammatory & Lab Markers	Total Leukocyte (WBC) Count	Kruskal–Wallis H	H = 2.41	—	0.299
Inflammatory & Lab Markers	Platelet Count (PLT)	Kruskal–Wallis H	H = 1.15	—	0.562
Inflammatory & Lab Markers	Aspartate Aminotransferase (AST)	Spearman Rank	rs = 0.082	[−0.054, 0.215]	0.236
Inflammatory & Lab Markers	Alanine Aminotransferase (ALT)	Spearman Rank	rs = 0.071	[−0.065, 0.204]	0.307
Inflammatory & Lab Markers	Creatine Phosphokinase (CPK)	Spearman Rank	rs = 0.042	[−0.094, 0.177]	0.542
Imaging & Outcomes	COVID-19 Disease Severity	Spearman / KW	rs = 0.576 (H = 81.3)	[0.478, 0.660]	<0.001
Imaging & Outcomes	Chest CT Lung Involvement	Kruskal–Wallis H	H = 15.02 (rs = 0.214)	[0.081, 0.340]	0.002
Imaging & Outcomes	ICU Admission Required	Mann–Whitney U	U = 1863.0	—	<0.001
Imaging & Outcomes	Length of Hospital Stay (LOS)	Spearman Rank	rs = 0.289	[0.159, 0.409]	<0.001
Imaging & Outcomes	In-Hospital Mortality	Mann–Whitney U	U = 1761.5	—	<0.001

Note: rs: Spearman rank-order correlation coefficient; 95% CI: 95% confidence interval for correlation coefficients calculated via Fisher's z-transformation; U: Mann–Whitney U test statistic for binary group comparisons; H: Kruskal–Wallis H test statistic for multi-group comparisons. Bold *P*-values indicate statistical significance (*p* < 0.05). All analyses are grounded in the complete cohort of *n* = 210 patients with verified serum IL-6 measurements.

Serum IL-6 levels showed statistically significant positive correlations with patient age (Spearman’s correlation coefficient, rₛ = 0.390; 95% confidence interval [CI]: 0.269 to 0.499; *p* < 0.001), length of hospital stay (rₛ = 0.289; 95% CI: 0.159 to 0.409; *p* < 0.001), and systemic inflammatory biomarkers, including CRP (rₛ = 0.238; 95% CI: 0.105 to 0.362; *p* < 0.001), troponin (rₛ = 0.201; 95% CI: 0.067 to 0.328; *p* = 0.003), ESR (rₛ = 0.177; 95% CI: 0.043 to 0.306; *p* = 0.010), and LDH (rₛ = 0.171; 95% CI: 0.036 to 0.299; *p* = 0.013).

Patients with pre-existing comorbidities had significantly higher serum IL-6 levels than patients without the corresponding comorbidities. Significant associations were observed for hypertension (Mann–Whitney U = 3,719.5; *p* < 0.001), diabetes mellitus (Mann–Whitney U = 2,873.5; *p* < 0.001), cardiovascular disease (Mann–Whitney U = 2,278.0; *p* < 0.001), and chronic kidney disease (Mann–Whitney U = 811.5; *p* = 0.007).

A significant inverse correlation was observed between serum IL-6 levels and baseline peripheral oxygen saturation (SpO₂) (rₛ = −0.398; 95% CI: −0.506 to −0.278; *p* < 0.001). In addition, patients with lymphopenia had significantly higher serum IL-6 levels than patients without lymphopenia (Mann–Whitney U = 4,310.5; *p* = 0.044). In contrast, no statistically significant associations were observed between serum IL, smoking status, acute symptoms smoking status, acute symptoms such as fever, cough, or dyspnea, anemia, total leukocyte count, platelet count, aspartate aminotransferase, alanine aminotransferase, or creatine phosphokinase (all *p* > 0.05; Table 6).

The extent of pulmonary involvement on chest CT was positively associated with serum IL-6 levels (rₛ = 0.214; 95% CI: 0.081 to 0.340; *p* = 0.002). Differences in IL-6 levels across CT involvement categories were also statistically significant (Kruskal–Wallis H = 15.02; *p* = 0.005). Furthermore, serum IL-6 levels progressively increased across the disease-severity categories from mild to critical disease (rₛ = 0.576; 95% CI: 0.478 to 0.660; *p* < 0.001; Kruskal–Wallis H = 81.30; *p* < 0.001).

Patients who required ICU admission had significantly higher median serum IL-6 levels than those managed in general medical wards (Mann–Whitney U = 1,863.0; *p* < 0.001). Similarly, non-survivors had significantly higher serum IL-6 levels than survivors (Mann–Whitney U = 1,761.5; *p* < 0.001).

## Discussion

SARS-CoV-2 (severe acute respiratory syndrome coronavirus 2) is a virus that caused COVID-19 (Coronavirus disease 2019), has resulted to noteworthy worldwide morbidity and mortality meanwhile its appearance in late December of 2019. First patients reporting the usual course of infection of SARS-CoV-2 explained a virus-related pneumonia giving as fever and an increasingly severe cough.^26^^,^^27^ As the start of the COVID-19 disease there have been numerous investigations that targeted to advance the forecast for the clinical progression of the virus. A number of biochemical factors were chose and evaluated as likely predictors of severity of the disease. The raised degree of IL-6, CRP, neutrophils, d-dimers, and a little lymphocyte amount have been related with COVID-19 severity forms.^28^^,^^29^ Through this current research, we investigated the medical characteristics of 210 COVID-19 patients. In the most cases, there were cough and fever as respiratory symptoms, myalgia, fatigue, chills, loss of appetite, shortness of breath and headache as non-respiratory signs. As demonstrated in work of Guan et al., 1099 patients with Covid-19 that confirmed laboratory from 552 hospitals in 30 provinces in China has revealed that Cough (67.8), Fatigue (38.1), Headache (13.6) are the highest clinical appearances of cases with Covid-19.^30^ Qin et al., have shown a research with record of clinical description of cases with Covid-19. The extreme frequent symptoms were fever (92.6%), Difficulty breathing (50.8%), expectoration (41.4%), fatigue (46.4%), dry cough (33.3%) and myalgia (21.4%).^31^^,^^32^ The above study results are fundamentally accordant with findings of this study. Consequently, medical doctors need to place significance on the medical signs of patients with doubted SARS-CoV-2 infection and increase their ability to separate SARS-CoV-2 infection from other illnesses. Mainly when the clinical appearances are non-respiratory symptoms, to diagnose cases imaging findings and laboratory indexes should be combined instantly, to aid quick intervention and avert SARS-CoV-2 transmission. We investigated the outcomes of research laboratory indicators and observed that the total number of white blood cells and the concentration of neutrophil in the blood of COVID-19 patients did not considerably vary, an important association was detected among lymphopenia and the amount of Interleukin-6, when the amount of lymphocyte cells in COVID-19 patients decrease, there is increase in Interleukin-6 level. In a study by Jurado and colleagues (2020), consistent with the present study, the results indicated a relationship between lymphopenia and the level of Interleukin-6.^33^

There was also a significant relationship observed between lactate dehydrogenase (LDH) and interleukin-6, such that in the group with abnormal LDH levels, a higher amount of interleukin-6 was present. In the study by Haroun and colleagues in 2023, similar to our study, LDH and interleukin-6 had a significant correlation with each other, and in addition, they were found to be among the most sensitive laboratory tests for predicting adverse outcomes in patients.^34^ Besides, we analyzed the variations in inflammation markers for example IL-6 and CRP in COVID-19 cases. We observed that CRP levels were greatly advanced in COVID-19 patients. Ayad et al., described that level of CRP was remarkably upper in COVID‐19 cases who died than in COVID‐19 survivors.^35^^,^^36^ Furthermore, levels of IL-6 in COVID-19 patients were meaningfully high. A research lead by Broman et al., have presented that the strongest marker of sever COVID-19 patients who hospitalized was IL-6.^37^ Also, a similar investigation demonstrated that level of IL-6 was related to degree of COVID-19 severity in COVID-19 cases. The results displayed that abnormal chest X-ray in patients was 4.72%. Regarding the chest CT scan of the patients, 91.5% showed involvement, with the highest frequency associated with moderate involvement (41.4%).^38^ The study by Mehrabi Nejad et al. indicates that certain chest CT findings at the time of admission, notably the PI score and PI density index, show strong potential as valuable instruments for forecasting patient outcomes.^39^ They report that less than 50% of COVID-19 patients with a history of cancer have classic COVID-19 imaging features. This renders it essential for radiologists to detect subtle, rare, or atypical chest CT characteristics in such patient groups.^40^ Therefore, our work provides an important laboratory basis for evaluating disease severity from patient IL-6 expression levels.

### Strengths, limitations, and future directions

A major strength of this study is the comprehensive evaluation of baseline serum IL-6 levels in conjunction with a wide array of clinical, paraclinical, and prognostic endpoints (Table 6). The demonstrated associations between elevated IL-6 concentrations and severe disease phenotypes, profound hypoxemia, ICU admission, and in-hospital mortality provide valuable real-world evidence for early risk stratification in COVID-19 patients.

Nevertheless, several methodological limitations should be acknowledged:

Study Design and Causality: Due to the cross-sectional design and single-point baseline measurement upon Emergency Department presentation, temporal or causal relationships between IL-6 dynamics and disease progression cannot be definitively established. Prospective cohorts with serial IL-6 measurements from admission to discharge are warranted to track its prognostic kinetics over time.

Confounding Treatments: Although blood samples were obtained upon admission to minimize the confounding effect of in-hospital therapeutic alterations, the potential influence of pre-admission medications (such as outpatient corticosteroids, antivirals, or immunomodulators) and the exact duration from symptom onset to presentation could not be fully controlled.

Single-Center Setting: This study was conducted at a single medical center (Shahid Beheshti Hospital, Kashan). Although the sample size (*N* = 210) yielded robust statistical power, large-scale multicenter prospective studies across ethnically diverse cohorts are recommended to confirm the generalizability of our findings.

## Conclusion

Increased serum Interleukin-6 (IL-6) concentrations demonstrate a close correlation with various key measures among COVID-19 patients such as prolonged hospitalization duration, higher age, decreased oxygen saturation, lymphopenia, elevated LDH and CRP, increased involvement on the CT scan, severe disease, ICU admission, and increased risk of mortality. Therefore, the tracking of IL-6 may become a useful predictor of many prognostic parameters among epidemic periods of COVID-19.

## Author contributions

Methodology and writing–original draft preparation: H.D. Formal analysis: H.R.G. conceptualization and methodology: Z.S. Supervision and project administration: Z.S. and A.A. Visualization: H.D. and K.E. Original draft preparation, writing and editing: A.A. and J.A.M. All authors have read and agreed to the published version of the manuscript.

## Ethics statement

Protocol of the study was studied and accepted by the Research Ethics Committee of Kashan University of Medical Sciences (ethics code: IR.KAUMS.MEDNT.REC.1401.029). Earlier the data assortment, the investigation purposes and protocol of study were clarified for each the members and informed consent was written and achieved.

## Consent

All authors are aware of participation and publication.

## Data availability

All data that support the findings of this article are fully contained within the published article (tables, figures, and text) and may be used without restriction for non-commercial research and educational purposes. No additional datasets were generated. Researchers who require further clarification or deidentified source data should contact the corresponding authors (Zahra Soleimani, Atena Abed), who will provide the information upon reasonable request.

## Ethics declaration

Written informed consent to take part in the study and to publish the article has been obtained from all participants or their legal representatives. The privacy rights of participants have been observed.

This study was performed in compliance with relevant laws, regulatory frameworks and guidelines where the research took place.This study was approved by the Institutional Ethics Committee of Kashan University of Medical Sciences.(Approval No. IR.KAUMS.MEDNT.REC.1401.029).

## Declaration of competing interest

The authors declare that there are no financial or personal relationships with any individuals or organizations that could inappropriately influence or bias the work reported in this manuscript.
